# 58 Achieving < 2cc/kg/%TBSA - A Restrictive Adjusted Ideal Body Weight Resuscitation Formula with Fresh Frozen Plasma

**DOI:** 10.1093/jbcr/irad045.032

**Published:** 2023-05-15

**Authors:** Steven Kahn, Bryttin Boyde, Keisha ONeill, Kathleen Hollowed, Elizabeth Halicki

**Affiliations:** The South Carolina Burn Center/Medical University of South Carolina, Charleston, South Carolina; The South Carolina Burn Center/Medical University of South Carolina, Charleston, South Carolina; South Carolina Burn Center at MUSC, Summerville, South Carolina; South Carolina Burn Center at MUSC, Charleston, South Carolina; South Carolina Burn Center @ MUSC, Charleston, South Carolina; The South Carolina Burn Center/Medical University of South Carolina, Charleston, South Carolina; The South Carolina Burn Center/Medical University of South Carolina, Charleston, South Carolina; South Carolina Burn Center at MUSC, Summerville, South Carolina; South Carolina Burn Center at MUSC, Charleston, South Carolina; South Carolina Burn Center @ MUSC, Charleston, South Carolina; The South Carolina Burn Center/Medical University of South Carolina, Charleston, South Carolina; The South Carolina Burn Center/Medical University of South Carolina, Charleston, South Carolina; South Carolina Burn Center at MUSC, Summerville, South Carolina; South Carolina Burn Center at MUSC, Charleston, South Carolina; South Carolina Burn Center @ MUSC, Charleston, South Carolina; The South Carolina Burn Center/Medical University of South Carolina, Charleston, South Carolina; The South Carolina Burn Center/Medical University of South Carolina, Charleston, South Carolina; South Carolina Burn Center at MUSC, Summerville, South Carolina; South Carolina Burn Center at MUSC, Charleston, South Carolina; South Carolina Burn Center @ MUSC, Charleston, South Carolina; The South Carolina Burn Center/Medical University of South Carolina, Charleston, South Carolina; The South Carolina Burn Center/Medical University of South Carolina, Charleston, South Carolina; South Carolina Burn Center at MUSC, Summerville, South Carolina; South Carolina Burn Center at MUSC, Charleston, South Carolina; South Carolina Burn Center @ MUSC, Charleston, South Carolina

## Abstract

**Introduction:**

Fresh frozen plasma (FFP) is used as an adjunct in burn resuscitation as it decreases endothelial cell permeability by restoring the glycocalyx. The authors have previously demonstrated a reduction in fluid administration using adjusted ideal body weight (AIBW) index to adjust for avascular adipose tissue with an FFP rescue. Since publication, the authors have further restricted the fluid administered in this protocol and hypothesize that this iteration results in further reductions without an increase in clinically significant acute kidney injury (AKI) and need for dialysis.

**Methods:**

A retrospective review of burn patients with 2^nd^/3^rd^ degree burns of 20% TBSA admitted between 6/20-5/22 at a single academic burn center was performed. Demographics and injury characteristics were tracked along with ventilator free days, volume/type of fluids administered during resuscitation, UOP, max creatinine (Cr) in the 1^st^ 72 hrs post injury, AKI requiring HD, length of stay (LOS), LOS O/E ratio (using NBR data), and death. Data was reported in medians + IQR. Subjects were resuscitated with 2mL/kg/%TBSA LR using an AIBW formula with FFP. AIBW index = ideal body weight + 0.3 [actual body weight – ideal body weight].

Those with 30-50% TBSA burns received 1 U FFP on admission; those with >50% TBSA had 2 U FFP. Starting hourly rate was calculated with 2 cc/AIBW in kg/%TBSA as a default, while 3 cc was used if mostly 3^rd^ degree injury + inhalation. Nursing driven titration was done by 10-20% per hr based on deviation from target UOP of 30cc/hr. For very obese or small patients, the UOP goal was reset to 0.5cc/AIBW/hr by MD. If oliguric for 2 hrs, patients received 1-2 units of FFP as rescue therapy.

**Results:**

Twenty-three patients were included in the study, and 10 received FFP during resuscitation. Burn size was 33% TBSA (IQR:27,38), and 3rd degree burn was 8% (IQR:3,15). Three subjects had inhalation injury, and 2 were resuscitated with 3cc/kg /%TBSA. Median fluid volume given over 24 hrs was 1.653 (IQR:1.022,2.358) cc/kg/%TBSA (including FFP). UOP was 0.67 cc/kg/hr (IQR:0.397,1.064). Ventilator free days was 29 (IQR: 24,30). One received a tracheostomy; 3 had escharotomies. Three (13%) patients had elevated Cr < 72 hours post injury (max creatinine- 1.7 mg/dL). LOS was 32 days (IQR:19,46) or 1.105 days/%TBSA. Median expected LOS was 88.5 days or 2.6 days/%TBSA based on NBR data. LOS O/E ratio was 0.42. Two patients died (9%).

**Conclusions:**

Despite the single-arm, retrospective nature, this study suggests that patients who were treated with a restrictive AIBW formula plus FFP received less total fluid than the previously published iteration of the formula (1.63 vs 3.3cc/kg/%TBSA), and far less than the traditional Parkland formula. Even with reduced fluids, no one had a clinically significant AKI. Preliminary data suggests this protocol is safe and effective, but additional prospective study is necessary.

**Applicability of Research to Practice:**

Treat burn shock and prevent over resuscitation

